# Microarray Meta-Analysis and Cross-Platform Normalization: Integrative Genomics for Robust Biomarker Discovery

**DOI:** 10.3390/microarrays4030389

**Published:** 2015-08-21

**Authors:** Christopher J. Walsh, Pingzhao Hu, Jane Batt, Claudia C. Dos Santos

**Affiliations:** 1 Keenan and Li Ka Shing Knowledge Institute of Saint Michael’s Hospital, Toronto ON M5B 1W8, Canada; E-Mails: chrisj.walsh@mail.utoronto.ca (C.J.W.); batt.jane@utoronto.ca (J.B.); 2 Institute of Medical Sciences and Department of Medicine, University of Toronto, Toronto ON M5B 1W8, Canada; 3 Department of Biochemistry and Medical Genetics, University of Manitoba, Winnipeg R3E 0J9, MB, Canada; E-Mail: pingzhao.hu@umanitoba.ca

**Keywords:** microarray platform, meta-analysis, normalization, biomarker

## Abstract

The diagnostic and prognostic potential of the vast quantity of publicly-available microarray data has driven the development of methods for integrating the data from different microarray platforms. Cross-platform integration, when appropriately implemented, has been shown to improve reproducibility and robustness of gene signature biomarkers. Microarray platform integration can be conceptually divided into approaches that perform early stage integration (cross-platform normalization) *versus* late stage data integration (meta-analysis). A growing number of statistical methods and associated software for platform integration are available to the user, however an understanding of their comparative performance and potential pitfalls is critical for best implementation. In this review we provide evidence-based, practical guidance to researchers performing cross-platform integration, particularly with an objective to discover biomarkers.

## 1. Introduction

The discovery of highly-reliable biomarkers from high-dimensional microarray data is an important goal in molecular medicine, with wide-ranging clinical applications. Potential roles for biomarkers include early detection of disease in healthy individuals, disease classification, prognosis, prediction of response to therapy, and as surrogate outcomes in clinical trials [1]. The ideal biomarker is inexpensive, robust, easily interpretable, well-validated, and clinically useful (e.g., improving prognosis or choice of therapy) compared to current standards of practice, meaning that the result is “actionable, leading to patient benefit” [1]. Publicly-available microarray data has vast potential to serve as a source of biomarker discovery as there is an enormous quantity of existing gene expression data [2,3]. At the present time, the Gene Expression Omnibus, a repository of array- and sequence-based expression data, currently contains 1,413,278 samples performed on 14,346 platforms [4]. The most widely known of these platforms include the Affymetrix GeneChips (*in situ* synthesized oligonucleotide microarray) and the Illumina high-density bead arrays [5]. While other types of microarrays exist, such as protein and microRNA [6,7], this review will focus on integration of gene expression data from multiple cDNA microarray platforms as it relates to the discovery of gene signatures that may serve as biomarkers for clinical applications. The integration of multiple data types (e.g., transcriptomic and proteomic data) has been proposed [8], however this is also beyond the scope of our paper.

While microarrays measure the expression of thousands of genes simultaneously, it is expected that only a small subset of the genes will be associated with the clinical or biological outcome of interest. This subset of genes, often termed a “gene signature” or “prognostic signature”, has a collective expression pattern that is unique to the outcome of interest and thus has potential to function as a biomarker [9]. The gene signature is typically composed of far fewer number genes (often less than 100 genes) than that on a microarray chip (often more than 20,000 genes) making it feasible for further study using approaches such as quantitative RT-PCR. Point of Care (POC) devices that rely on transcriptional signatures are progressively gaining momentum as diagnostic tools for routine use in the clinical setting, resulting from their practical and affordable application making this approach highly accessible as cheaper diagnostic kits [10,11].

Biomarkers for the monitoring of disease activity of POC are currently lacking. A number of published gene signatures validated using independent samples have been shown to serve as significant predictors of clinical outcome [12,13,14,15]. However, the development of prognostic signatures that are robust and stable (e.g., the same biomarkers are identified in both discovery and validation sets) [16] has proven challenging [17,18,19]. In Section 3, we will discuss recent examples of promising transcriptomic biomarkers for disease diagnosis and prognosis that have been identified using meta-analysis approaches.

Published prognostic gene signatures derived from internal validation often show little overlap with genes identified by other study groups [15]. Potential causes of small reproducibility include differences in sample collection methods, processing protocols, and microarray platforms, patient heterogeneity, and small sample sizes [12]. Due to the difficulty of acquiring samples, particularly from human tissue and the associated costs, microarray experiments from single-institution patient cohorts are often composed of small sample sizes. Predictive models trained on the gene signatures identified from these smaller-sized individual studies are less robust [15,20]. Michiels *et al.* [21] re-analyzed data from nine studies predicting cancer prognosis and found an unstable misclassification rate for the gene signature (defined as the 50 genes for which expression was most highly correlated with outcome) using training sets derived using a re-sampling approach, with performance increasing as the size of the training set increases*.*

Integration of multiple microarray data sets has been advocated to improve gene signature selection [22]. Increasing sample sizes increases the statistical power to obtain a more precise estimate of integration of (differential) gene expression and to assess the heterogeneity of the overall estimate, as well as to reduce the effects of individual study-specific biases [23,24,25,26]. Meta-analysis is most commonly applied for the purpose of detecting differentially-expressed (DE) genes [27] which may serve as a candidate gene signature or be used as features in classification models or classifiers to further refine a clinically useful gene signature [28]. Supervised classification techniques (also known as prediction analysis or supervised machine learning) are the most commonly used methods in microarray analysis that lead to identification of clinically-useful biomarkers (*i.e.*, gene signatures providing improved discrimination between two or more patient groups) [27]. Classification methods for gene signature selection are beyond the scope of this article and have been reviewed elsewhere [29].

## 2. Integrative Transcriptomic Data Analysis

**Figure 1:**
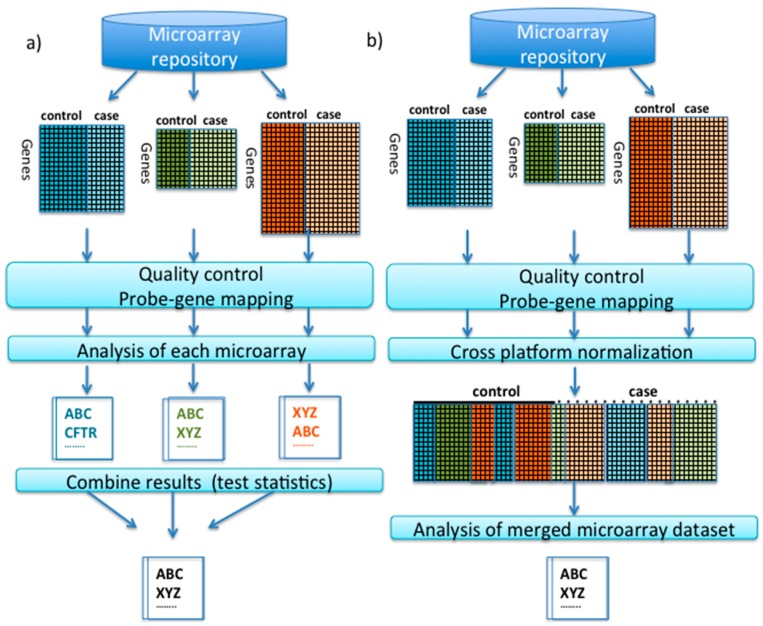
Outline of two microarray integration methods: (**a**) meta-analysis (“late integration”). Individual case-cohort microarray studies are pre-processed and each study is used to identify ranked gene lists which are then combined in the final step; (**b**) Cross-platform merging and normalization (“early integration”). After pre-processing of individual studies, a single unified case-cohort dataset is generated (“clustered” into cases and cohorts, indicating removal of batch to batch variation) and in this example, used to identify a ranked gene list.

Two fundamental approaches to combine the information of multiple independent microarray studies from different platforms (termed “integrative analysis” [23]) are meta-analysis and cross-platform normalization (also termed “merging”). A conceptual framework by Hamid *et al.* [22] classifies microarray meta-analysis as “late stage” data integration as it combines the final statistic results from different studies, whereas cross-platform normalization integrates data at the “early stage”. Application of these approaches necessitates that all of the included studies are testing the same hypothesis and/or performed under comparable conditions or treatments [2,30,31]. While the degree of similarity that is required between “suitably similar” datasets still remains to be determined, cross-platform integration for the purpose of biomarker discovery is most appropriate using relatively homogenous datasets selected to answer well-defined questions [32]. Early or late stage integration of data can be used regardless of the biological question (e.g., differential expression analysis or class prediction). The overall principle of these two approaches is summarized in Figure 1.

### 2.1. Pre-Processing and Quality Control Prior to Integrative Analysis

Ramasamy *et al.* [24] identified key issues and steps for performing a meta-analysis including identifying suitable microarrays, pre-processing and preparing individual datasets, selection of meta-analysis method, and interpretation of results. A systematic review of microarray meta-analysis studies in the literature has found that the criteria to include or exclude microarray studies is mostly subjective and *ad hoc* and remains an open question in the field [27]. Two critical pre-processing steps we will highlight here are (i) removing arrays with poor quality and (ii) determining the relationships between probes and genes. Identifying microarrays of poor quality is essential prior to integrative analysis because inclusion of poor quality studies may reduce statistical power and adversely affect the outcome of meta-analysis [27,33]. There are a number of quality assessment packages available for Bioconductor, including Simpleaffy [34] and affyPLM [35] for Affymetrix. The MetaQC package provides six quality control measurements to identify problematic studies across multiple platforms for further assessment of causes of lower quality to determine their exclusion from meta-analysis [36,37].

Another important pre-processing step is ascertaining which probes represent a given gene within and across the different microarray platforms. The relationship between probes and genes may be determined by mapping probes to the gene using sequence-matched datasets or using gene-level identifiers such as Entrez Gene ID available in the annotations packages in R/Bioconductor [38] to unify the microarray datasets. Sources of high-quality probe re-annotation include alternative chip definition files (CDFs) for Affymetrix [39] and ReMOAT (Re-annotation and Mapping for Oligonucleotide Array Technologies) and its associated annotation packages in R/Bioconductor for Illumina [40]. Only genes that are present across the different platforms being integrated will remain for further analysis, while those absent in one or more platforms will be “lost”, reflecting the tradeoff between increasing sample size and power *versus* decreasing the number of genes analyzed [32]. Co-inertia analysis, a multivariate analysis method that describes the common trends or co-relationships between datasets of two conditions, has been applied to determine the loss of information incurred by reducing the number of genes to the subset common to different platforms [41]. Imputation of gene expression present in some datasets, but not others, to allow these genes to be part of predictive models has been proposed [42].

If multiple probes match a single gene, selecting the probe with the highest interquartile range (IQR) has been recommended [43]. Genes with low mean expression across most studies are typically filtered out prior to meta-analysis. Turnbull *et al.* [32] applied relatively strict filter thresholds for their microarray integration analysis based on a prior study that found genes with low or intermediate expression have poorer inter-platform reproducibility than highly-expressed genes [17,44]. Furthermore, incorporation of a quality measure based on detection *p*-values estimated from Affymetrix arrays into the study-specific test statistics within a meta-analysis of two Affymetrix array studies using an effect sized model produced more biologically meaningful results than an unweighted model [25,45].

### 2.2. Meta-Analysis

In the meta-analysis approach, each experiment is first analyzed separately and the results of each study are then combined. Meta-analysis methods that combine primary statistics (e.g., *p*-values or effect sizes) require the use of raw gene expression data whereas secondary statistics rely only on ranked lists of genes. Popular methods for meta-analysis mainly combine one of three types of statistics: *p*-value [46], effect size [47], and ranked gene lists (“rank aggregation”) [27,33,48]. Ranked lists of genes produced for each study (e.g., ranked by order of *p*-value for DE of each gene) have been aggregated into a single gene ranking (“consensus”) using a number of methods including the rank product method [48].

A number of methods have been developed to test the statistical significance of results based on combining *p*-values from each study including Fisher’s method, Stouffer’s method, minP, and maxP. Fisher’s method sums log-transformed *p*-values, whereas Stouffer’s method sums inverse-normal-transformed *p*-values, to combine statistical significance across studies. The minP method takes the minimum *p*-value from combined studies, whereas the maxP method takes the maximum of the combined *p*-values. Rhodes *et al.* [49] published one of the first papers to combine *p*-values from individual studies of DE gene expression using Fisher’s method which found improved statistical significance using the combed analysis compared to individual studies.

Combined effect size to generate an estimate of the overall effect size and its confidence interval is frequently used in meta-analysis of clinical research data. Choi *et al.* [47] described one of the first methods to combine effect sizes using a random-effects modeling approach for combining datasets from individual studies of two groups to form an overall estimate of the weighted effect size. The effect size was measured by the standardized mean difference obtained by dividing the difference in the average gene expression between the treatment and control groups by a pooled estimate of standard deviation. The effect size was used to measure the magnitude of treatment effect in each study and a random effects model was used to incorporate inter-study variability.

Meta-analysis methods have been categorized based on the hypothesis settings that gene biomarkers are differentially expressed “in all studies” (HS_A_), “in the majority of studies” (HS_r_), or in “one or more studies” (HS_B_) [33,50]. In Fisher’s, Stouffer’s, and minP method, an extremely small *p*-value in one study likely meets criteria for statistical significance; thus, it detects DE in “in one or more studies” (HS_B_), whereas the maxP or rank product method tends to detect gene biomarkers DE in “all studies” (HS_A_).

The choice of the statistical meta-analysis method is selected based on the biological purpose of the analysis. A gene serving as a biomarker from a meta-analysis is expected to show concordant biological effects across all or most experiments for a given condition derived from relatively homogenous sources (e.g., up-regulation of a gene predicting risk of lung cancer detection from lung epithelium biopsied from a cohort of smokers *versus* healthy non-smokers) [51]. While detecting biomarkers DE in all studies seems an ideal goal, it can be too stringent when the number of samples is large, increasing the heterogeneity of experimental, platform, or biological samples [50]. Meta-analysis methods detecting DE in the majority of samples (HS*_r_*) are generally recommended as they provide robustness and detection of relevant signals across the majority of samples [33]. Song and Tseng [52] proposed a robust order statistic, rth ordered p-value (rOP), which tests the alternative hypothesis that there are significant *p*-values in at least a given percentage of studies. This method detects biomarkers DE in the majority of studies (e.g., >70% of studies) based on a user-specific threshold of studies.

#### 2.2.1. Comparison of Meta-Analysis Methods

Several comparative studies systematically comparing meta-analysis methods for microarray data have been previously published [33,53,54]. Chang *et al.* [33] benchmarked the performance of six *p*-value combination methods (Fisher, Stouffer, adaptively weighted Fisher, minP, maxP, and rOP), two combined effect size methods (fixed effects and random effects) and four combined ranks methods (RankProd, RankSum, product of ranks, and sum of ranks). The 12 meta-analysis methods were categorized into three hypothesis settings (candidate markers DE in “all” [HS*_A_*], “most” [HS*_r_*], or “one or more” [HS_B_] studies) based on their strengths for detecting DE genes. They then applied four statistical criteria to the assessment of each meta-analysis method: (1) detection capability (the number of DE genes detected); (2) biological association (degree of association between DE list with predefined genes from pathways related to the disease), stability (randomly splitting the data and comparing results of the two-meta-analyses) and robustness (effect of including an outlying irrelevant study to the meta-analysis).

Among the methods based on HS_A_ setting, the maxP performed the worst based on their four criteria and the investigators recommend that it be avoided. Rank product method had improved performance but weaker detection capability. The two methods that tended to detect DE in the majority of samples were the Random Effect Model (REM) and the rth order *p*-value (rOP). rOP outperformed REM based on stronger biological association and detection capabilities, but this was achieved at the expense of diminished stability and robustness.

It is important to note that differentially-expressed genes determined by combing *p*-values or ranks obtained by two-sided hypothesis testing may result in genes with discordant DE across two-class outcomes which can be difficult to interpret [27]. Wang *et al.* [37] have proposed one-sided correction of *p*-values to guarantee identification of DE genes with concordant DE direction.

#### 2.2.2. Association of Meta-Analysis Method to Outcome Variable

The objective and type of outcome types (e.g., two-class, multi-class, survival) [24] will govern the choice of both the test statistic (*t*-statistic, *F*-statistic, log-rank statistic) and the meta-analysis method (combing *p*-values, effect sizes, or ranks). Methods combing effect sizes (standardized mean differences or odds ratios) are appropriate for combining two-class outcomes. Meta-analysis of expression studies with continuous outcomes (e.g., using regression or correlation coefficients) and survival outcomes (based on log-rank statistics) have typically been performed using combined *p*-values [50,55] and can be performed using the MetaDE package [37]. To capture concordant expression patterns for multi-class outcomes, Lu *et al.* [52] have applied multi-class correlation (min-MCC) because the *F*-statistic has been found to frequently fail to capture concordant patterns of gene expression.

### 2.3. Cross-Platform Normalization

Cross-platform normalization (also termed “data merging” [23]) considers all data from experiments across different microarray platforms as a single data set from the same experiment. Direct integration of data sets performed on different microarray platforms may introduce undesirable batch effects due to systematic multiplicative biases [23,32,56]. The level of difficulty present to combine multiple datasets has been termed “dataset complexity” [53]. For example, integrating different Affymetrix platforms is less complex to analyze by meta-analysis or cross platform normalization than datasets performed across very different platforms. Studies using low complexity datasets, mainly from the Affymetrix platform, have directly merged the studies to construct a gene signature [41,57,58,59].

Cross-platform transformation and normalization methods have been developed with an aim to remove the artifactual differences between data from different microarray platforms while preserving the underlying biological differences between conditions. This step is essential, as non-biological differences (“batch effects”) in the gene signature discovery data can obscure real biological differences found between clinical groups.

Early attempts at cross-platform merging applied straightforward transformation methods of location and scale (mean and variance) to process the gene expression data from different studies. Batch mean centering [56] is a simple transformative method that standardizes the expression of each gene to have the same center (mean expression). Probe sets can be further transformed to have the same variance or distributions on different platforms [60,61]. While these methods are relatively easy and intuitive, the batch mean centering method has been shown to have only marginal improvement compared to uncorrected data for cross-platform integration of Illumina and Affymetrix data [32]. Probability of expression (POE), a model-based transformation that is estimated based on a method that adopts an underlying mixture distribution that transforms each data value into range [−1,1] has been used for cross-platform merging based on a unified scale as an alternative to using gene-specific summaries [62,63]. While this transformation has been applied for identifying meta-signatures, it has been found to be difficult to compare to other normalization methods [26].

Over the past decade, a number of more complex cross-platform normalization methods have been published and their performance has been compared in several studies [2,32]. Four cross-platform normalization methods found to be generally effective in a comparative review by Rudy and Palafer [2] are the Empirical Bayes (EB) method, known as Combat [64]; the Cross-Platform Normalization (XPN) method [26], Distance Weighted Discrimination (DWD) [65], and the Gene Quantiles (GQ) method developed as part of the WebArrayDB service [66]. Of these four programs, the authors favour DWD and XPN, while the comparative analysis of cross-platform normalization methods on clinical datasets by Turnbull *et al.* [32] favoured Combat and XPN. We will discuss the results of these comparative analyses in more detail in the following Section 2.3.1.

The Distance Weighted Discrimination method, like Support Vector Machines (SVM), is a margin-based classification method that was developed to improve performance over the latter method. Essentially, SVM finds a hyperplane that separates the two classes (*i.e.*, each systematic bias) to maximize the minimum distance of all the data on the hyperplane (the margin). However SVM has data pile-up problems along the margin which have been improved by modifying the margin to maximize the sum of the inverse distance in DWD [67]. DWD adjusts the microarray data by projecting the different batches onto the hyperplane, finding the batch mean and then subtracting out the plane multiplied by this mean.

Combat, an empirical Bayes method, estimates parameters that represent the batch effects by pooling information across genes in each batch to shrink the batch effect parameter toward the overall mean of the batch effect estimates across genes [64]. The data are then transformed to remove the effects of the different batch effect parameters across platforms. Combat is performed using either a parametric prior method or a non-parametric method based on the prior distributions of the estimated parameters [68].

Unlike the gene-wise linear approaches of DWD and Combat, the cross-platform normalization (XPN) method developed by Shablin *et al.* [26] seeks to borrow information across genes and samples via linked row and column clusters in a two-step procedure. First, K-means clustering is used to find blocks of similar genes and samples across the platforms. This approach is robust to the number of row (K) and column (L) clusters. Then, within each block the data is normalized between platforms within this block. The normalized values obtained over multiple clustering performed over repeated runs is then averaged to better capture the data structure.

#### 2.3.1. Comparison of Cross-Normalization Methods

A comparative analysis of cross-platform normalization methods by Rudy and Valafar [2] found the DWD classification method to provide effective batch adjustment for microarray data [67] and to be the most robust to variation in treatment group sizes between the platforms with the least loss of treatment information (lower underdetection), while XPN showed the greatest inter-platform concordance [2]. Turnbull *et al.* [32] also found that XPN had the highest inter-platform concordance. However, they found that DWD removed not only the platform specific systematic bias, but also relevant biological variability between samples (reduced inter-sample variance), while Combat and XPN preserved this biological signal (slightly increased inter-sample variance) while appropriately correcting the platform specific bias (reduced inter-platform variance). The drawback of DWD to “over-normalize” by removing all systematic expression differences between two datasets, including the relevant biological variability has been cautioned by other authors, prompting development of newer methods [60]. Although Combat and XPN have been found to perform well in previous analyses, the user must be cautious when applying this method to datasets that are unbalanced (e.g., different subtypes within each of the batches) as these methods will not be able to distinguish batch effects from biologically-relevant signals [42].

One limitation of some existing cross-platform normalizing methods is that they can only be applied to two batches at a time. While cross-platform normalization steps can be chained together, the effect of these multiple normalization steps or which chaining method is still unclear [60].

#### 2.3.2. Software and Websites Implementing Microarray Meta-Analysis and Cross-Platform Merging/Normalization

Software, including packages in R/Bioconductor and websites allowing users to implement microarray meta-analysis and cross-platform merging and normalization methods are listed in Table 1. Different experiments from multiple different arrays can be directly merged from the CEL files simultaneously using several packages implemented in R [69] including inSilico Merging [70], the CONOR [2], and virtualArray [71]. The inSilico Merging package implements XPN, DWD, and Combat, and the package CONOR additionally implements the GQ method. The virtualArray package allows cross-platform normalization using empirical Bayes methods (default) or the user may select one quantile discretization, normal discretization normalization, gene quantile normalization, median rank scores, quantile normalization, or mean centering [71]. This batch effect removal step can be supervised allowing the user to specify samples into groups based on platform as well as other attributes (e.g., cell type). Before the combined expression data undergoes cross-platform normalization, the data must be transformed to a common scale (e.g., log2) and resolution (e.g., 12, 14, 16, or 20 bit) [71]. As with meta-analysis, low expression and low variance genes are typically filtered out.

**Table 1:** List of software and websites for performing microarray meta-analysis.

Microarray Meta-Analysis (Command Line Packages)		
**Software Name**	**Language**	**Features**
metaDE (metaOmics)	R	Implements 12 major meta-analysis methods [37]
MAMA	R	Implements combined effect size, combined *p*-values, combined ranks
metaMA	R	Implements combined moderated effect size, combined *p*-values
metaGEM	R	Implements combined effect size, combined *p*-values, vote counting [24]
metahdep	R	Effect size estimates particularly when hierarchical dependence is present
GeneMeta	R	Implements combined effect size [47]
OrderedList	R	Combine ranks with or without expression data
RankProd	R	Implements Product of Ranks method
RankAggreg	R	Aggregation of ordered lists based on the ranks using several different algorithms
Automated web applications for microarray meta-analysis/normalization		
**Software Name**		**Features and URL**
INMEX		Meta-analysis. Support for 45 microarray platforms for human, mouse rat. Combines *p*-values, effect sizes, rank order, others http://www.inmex.ca/INMEX/
Network Analyst		Meta-analysis. Combines *p*-values, effect sizes, rank order. Significantly altered genes are then presented within the context of protein-protein interaction networks. http://www.networkanalyst.ca/NetworkAnalyst/faces/home.xhtml
A-MADMAN		Affymetrix platform normalization using quantile distribution transformation http://compgen.bio.unipd.it/bioinfo/amadman/
MAAMD		Affymetrix meta-analysis http://www.biokepler.org/use_cases/maamd-workflow-standardize-meta-analyses-affymetrix-microarray-data
Microarray cross-platform merging/normalization (command line packages)		
**Software Name**	**Language**	**Features**
mergeMaid	R	Implements Probability of Expression transformation (POE) [62]
metaArray	R	Implements POE [62]
CONOR	R	Implements XPN, Empirical Bayes (EB), Quantile normalization (QN), Quantile discretization (QD), others [2]
VirtualArray	R	Implements EB, QN, QD, others [71]
inSilico Merging	R	Implements XPN, EB, DWD, others [23]
Automated Microarray Data Analysis v2.13	R	Implements. Allows analysis of Illumina, Affymetrix and Agilent.
XPN	R	Implements Cross Platform Normalization [26]
DWD	JAVA, R MATLAB	Implements Distance Weighted Discrimination method [65]
Combat	R	Implements Empirical Bayes methods [64]
PLIDA	MATLAB	Normalizes an arbitrary number of platforms [60]
metAnalyzeAll	R	Elastic net classifier [42]

### 2.4. Comparison of Meta-Analysis vs. Cross-Platform Normalization

Directly-merged microarray data (or applying cross-platform normalization) has been argued to have better performance than meta-analysis for the identification of robust biomarkers on the premise that “deriving separate statistics and then averaging is often less powerful than directly computing statistics from aggregated data” [57]. In a comparative study, Taminau *et al.* [23] found significantly more differentially-expressed genes using cross-platform normalization than meta-analysis. An additional advantage of cross-platform normalization is that it allows prediction models applied to a subset of studies to be applied across additional studies from other platforms [27]. While cross-platform normalization has been applied in multiple studies [72,73,74], it has less frequently been used in the literature compared to meta-analysis [2]. A recent comprehensive systematic literature review of studies applying microarray integration methods found that only 27% of the studies directly merged microarray data and this subset of studies were mostly performed on the same platform [27].

One major limitation of existing cross-platform normalization is that they require that every treatment group or sample type be represented on each platform to allow differentiation of treatment effects from platform effects. Furthermore, cross-platform normalization methods do not guarantee elimination of laboratory or batch effects across experiments and Rung and Brazma [3] have argued that microarray meta-analysis provides better control of between-laboratory heterogeneity, which can be estimated using Cochrane’s Q statistic and be correspondingly adjusted.

## 3. Promising Transcriptomic Biomarkers Identified Using Meta-Analysis Approaches

Sweeney *et al.* [75] recently identified a transcriptomic signature to improve discrimination of patients with sepsis (infection) from those with sterile inflammation using blood samples. Their work analyzed publicly-available gene expression datasets from 22 independent cohorts (composed of 2903 microarrays in total) and applied a meta-analysis strategy implementing both effect size and *p*-values of differential gene expression. The investigators identified 82 genes differentially expressed between sepsis and inflammation and then performed a greedy forward search to determine which combination of these 82 genes produced the best improvement of area under the curve (AUC) in their discovery datasets. This resulted in an 11-gene transcriptional signature that was applied to 15 independent validation cohorts and was found to improve discrimination of patients with infection from those with sterile inflammation compared to use of clinical data alone. This gene signature requires further validation using prospective cohorts, however its excellent discriminatory power in both the discovery and validation cohorts suggests that it is likely to become a useful clinical assay in the future.

Santiago and Potashkin [76] implemented a transcriptomic and network-based meta-analysis in NetworkAnalyst (Table 1) to identify potential key hub genes in the blood of patients with Parkinson’s disease (PD). Their analysis identified hepatocyte nuclear factor 4 alpha (*HNF4A*) and polypyrimidine tract binding protein 1 (*PTBP1*), as the most significant up- and down-regulated genes in blood samples from PD patients. The relative abundance of *HFN4A* mRNA was found to correlate with disease severity in PD and the results were validated using samples obtained from two independent clinical trials. The abundance of *HNF4A* and *PTBP1* mRNAs significantly decreased and increased, respectively, in PD patients during a 3-year follow-up period suggesting that these biomarkers may be useful for monitoring disease-modifying therapies for PD.

## 4. Confounding Adjustment

In the previously discussed cross-platform normalization approaches (Section 2.3), the major batch effect (the platform) is clearly identified (“supervised”), in distinction to other confounding adjustment methods such as using surrogate variable analysis (SVA) which detect “latent” (unknown) variables such as experimental variability or patient subgroups (e.g., breast cancer subtypes). It is important to account for possibly confounding (e.g., age or sex) or possibly predictive variables (e.g., smoking history) in addition to gene expression for building a gene signature. Additional categorical and continuous variables can be easily included along with the gene expression data using regression methods such as the elastic net penalty to fit a generalized linear model (GLM) [42]. These models can also be readily adapted for different outcomes such as categorical, continuous, and survival times. Cho *et al.* [77] developed a software program (rbsurv) to detect survival-associated genes based on the partial likelihood of the Cox model that allows adjustment of for risk factors in survival modeling.

Modelling confounding factors with variable selection in meta-analysis has recently been shown to improve robustness and sensitivity of DE gene detection [43] and inter-study concordance [78]. Chikina *et al.* [78] produced corrected differential expression lists using surrogate variables calculated using a modified version of SVA with improved inter-study agreement over uncorrected analysis. A two-class meta-analysis by Wang *et al.* [43] applied a random intercept model to account for confounding covariates in each single study analysis and combined *p*-values of the candidate biomarker list from each study using Fisher’s and maxP methods. Statistical approaches to allow synthesis of regression slopes in meta-analysis have been described [79] and applied to meta-analysis [51].

## 5. Conclusions

Gene signature discovery for prognostic and diagnostic purposes is improved with knowledgeable selection and appropriate application of integration methods on microarray data performed on multiple platforms. While no consensus for the best implementation of cross-platform integration is currently available, previous benchmarking and comparative analyses have established the strengths and limitations of many of the existing methods. The recent evidence suggesting improved performance of cross-platform normalization methods over meta-analysis may lead to an increasing proportion of studies in the literature implementing the former method. Further refinement of existing methods and development of new methods for cross-platform normalization and classification to exploit the vast quantity of microarray data currently available are expected. As elimination of platform specific bias becomes well-established with these methods, future studies addressing the performance of prognostic signature discovery in light of the existing *biological* heterogeneity will become a central focus.
